# Etiology of diarrheal hospitalizations following rotavirus vaccine implementation and association of enteric pathogens with malnutrition among under-five children in India

**DOI:** 10.1186/s13099-024-00599-8

**Published:** 2024-04-10

**Authors:** Tintu Varghese, James A. Platts Mills, R. Revathi, Sebastien Antoni, Heidi M. Soeters, Tondo Opute Emmanuel Njambe, Eric R. Houpt, Jacqueline E. Tate, Umesh D. Parashar, Gagandeep Kang

**Affiliations:** 1 The Wellcome Trust Research Laboratory, Division of Gastrointestinal Sciences, Christian Medical College, Vellore, India; 2 Division of Infectious Diseases and International Health, University of Virginia, Charlottesville, Virginia USA; 3 World Health Organization, Geneva, Switzerland; 4 World Health Organization Regional Office for South-East Asia, New Delhi, India; 5 Division of Viral Diseases, Centers for Disease Control and Prevention, Atlanta, Georgia USA

**Keywords:** Malnutrition, Children, Diarrhea, Enteropathogens, Molecular testing, Low resource settings, qPCR

## Abstract

Malnourished children are at higher risk of mortality and morbidity following diarrheal illness and certain enteropathogens have been associated with malnutrition in children. Very few studies have comprehensively looked at the etiology of diarrhea in malnourished children and most have used conventional diagnostic methods with suboptimal sensitivity. We used a highly sensitive molecular approach against a broad range of pathogens causing diarrhea and examined their association with malnutrition. In addition, we looked at the pathogen diversity of pediatric diarrhea, three years after the nationwide rotavirus vaccine introduction to understand the evolving landscape of pathogens, which is crucial for planning strategies to further reduce the diarrhea burden. Clinical details and diarrheal stool samples were collected from hospitalized children aged < 5 years from three sentinel sites in India for a period of one year. The samples were tested by qPCR for 16 established causes of diarrhea using TaqMan Array Cards. A total of 772 children were enrolled, from whom 482 (62.4%) stool specimens were tested. No specific pathogen was associated with diarrhea among children with acute or chronic malnutrition compared to those with better nutritional status. Overall, adenovirus was the leading pathogen (attributable fraction (AF) 16.9%; 95% CI 14.1 to 19.2) followed by rotavirus (AF 12.6%; 95% CI 11.8 to 13.1) and *Shigella* (AF 10.9%; 95% CI 8.4 to 16.4). The majority of diarrhea requiring hospitalization in children aged < 2 years could be attributed to viruses, while *Shigella* was the most common pathogen among children aged > 2 years. These data on the prevalence and epidemiology of enteropathogens identified potential pathogens for public health interventions.

## Introduction

Diarrheal disease is a principal cause of morbidity and mortality in children < 5 years of age in developing countries. Globally diarrheal illness accounts for an estimated 13.5% of childhood stunting [1], and children with acute malnutrition are three times more likely to die from diarrhea than children with better nutritional status [2]. Indian children carry a dual burden of malnutrition and diarrhea. As per the latest National Family Health Survey (NFHS) conducted in 2019, 35.5% of Indian children under-five years are stunted and 32.1% are underweight [3]. The association between malnutrition and diarrhea is considered bidirectional. Decreased dietary intake, malabsorption, and increased catabolism during diarrhea lead to malnutrition; whereas impaired immunity increases the chances of diarrhea in malnourished children [4]. Environmental enteropathy and enteric microbiome dysbiosis predispose malnourished children to severe diarrheal disease [5]. The negative influence of diarrhea on nutrition has recently been shown to be a pathogen-specific association [6]. Understanding the etiology and clinical presentation of diarrhea among malnourished children is important in case management and to prioritise control measures targeting specific pathogens. We aimed to describe the diarrheal phenotype in malnourished children, in addition to examining the relationship between nutritional status and any specific enteropathogens.

There have been a number of initiatives undertaken by the Indian government over the last few years to reduce diarrheal diseases, such as the 'Bharat Nirman’, ‘Swachh Bharat Abhiyan’, and ‘Total Sanitation Campaign', aimed at promoting sanitation, hygiene, safe drinking water and nutrition. In 2016, the introduction of rotavirus vaccines into the Universal Immunization Program (UIP) marked a major milestone in the fight against childhood diarrhea. A reduction in diarrhea has been reported since the implementation of these measures [7]. With the large-scale use of rotavirus vaccines, a change in the pattern of diarrheal pathogens has been reported worldwide [8–11], but few such data are available from India. Estimating the pathogen prevalence in the post-rotavirus vaccine era supports prioritization of interventions to control diarrhea. The World Health Organization (WHO) has coordinated the Global Rotavirus Surveillance Network (GRSN) since 2008 [12] which was later expanded to Global Pediatric Diarrhea Surveillance (GPDS) [11] to collect data on diarrheal admissions (watery diarrhea and dysentery) among under-five children at sentinel surveillance sites established across various countries including India. We used the samples and data collected through the three GPDS surveillance sites in India during 2019 to better understand (a) the pathogen profile for under-five diarrhea children in the post-rotavirus vaccine era, (b) whether pathogen attribution was associated with nutritional status, and (c) the diarrhea phenotype in malnourished children.

## Results

A total of 772 children hospitalized with diarrhea were enrolled from January 2019-December 2019 from the three sentinel sites, of whom 361/772 (46.8%) were stunted. Acute malnutrition status was available for 629 children, of whom 70 (11.1%) were acutely malnourished. 55.3% of children were males and 44.7% were females. Nearly 81% of the diarrheal hospitalizations were among children aged less than 24 months. The majority of children presented with acute diarrhea (99.7%), associated with vomiting (64.1%), and fever (59.8%). 17% had severe dehydration at the time of admission and 5.2% of children had dysentery. In almost all cases, children had access to improved drinking water and 90.7% had received at least one dose of the rotavirus vaccine. 482 (62.4%) stool specimens were tested by qPCR for 16 established etiological causes of diarrhea using a custom-designed TaqMan Array Card. Of the 482 samples tested, 251 (52%) were less than 12 months of age, 175 (36.3%) children were between 12 and 23 months of age, and 56 (11.6%) children were older than 2 years. 243 (50.4%) children had chronic malnutrition and 51 children out of 421 for whom acute malnutrition status could be assessed (12.1%) had acute malnutrition. The sociodemographic and clinical characteristics of the children are listed in Table 1.

**Table 1:** Demographic and clinical characteristics of study population

	Total children enrolled (N = 772)	Samples tested (N = 482)	Acute malnutrition (N = 51)	No Acute Malnutrition (N = 370)	Chronic malnutrition (N = 243)	No Chronic malnutrition (N = 239)
Gender						
Male	427 (55.3%)	260 (53.9%)	33 (64.7%)	191 (51.6%)	134 (55.1%)	126 (52.7%)
Female	345 (44.7%)	222 (46.1%)	18 (35.3%)	179 (48.4%)	109 (44.9%)	113 (47.3%)
Age						
< 6 months	179 (23.2%)	73 (15.1%)	6 (11.8%)	18 (4.9%)	23 (9.5%)	50 (20.9%)
7–11 months	217 (28.1%)	178 (36.9%)	24 (47.1%)	142 (38.4%)	107 (44.0%)	71 (29.7%)
12–23 months	228 (29.5%)	175 (36.3%)	14 (27.5%)	161 (43.5%)	93 (38.3%)	82 (34.3%)
24–35 months	100 (13.0%)	42 (8.7%)	5 (9.8%)	37 (10.0%)	15 (6.2%)	27 (11.3%)
36–59 months	48 (6.2%)	14 (2.9%)	2 (3.9%)	12 (3.2%)	5 (2.1%)	9 (3.8%)
Sentinel site						
Tirupati	260 (33.7%)	188 (39.0%)	8 (15.7%)	166 (44.9%)	125 (51.4%)	63 (26.4%)
Rohtak	311 (40.3%)	181 (37.6%)	21 (41.2%)	138 (37.3%)	93 (38.3%)	88 (36.8%)
Vellore	201 (26.0%)	113 (23.4%)	22 (43.1%)	66 (17.8%)	25 (10.3%)	88 (36.8%)
Acute diarrhea (< 7 days)	770 (99.7%)	480 (99.6%)	49 (96.1%)	370 (100%)	242 (99.6%)	238 (99.6%)
Dysentery*	25 (3.2%)	25 (5.2%)	3 (5.9%)	21 (5.7%)	13 (5.3%)	12 (5.0%)
Maximum diarrheal episodes in 24 h median [range]	8.00 [3.00, 30.0]	10.0 [3.00- 30.0]	7.00 [3.00- 20.0]	10.0 [3.00—30.0]	10.0 [3.00, 20.0]	8.00 [3.00, 30.0]
Diarrheal duration in days; mean [SD]	2.91 (4.53)	3.03 (5.65)	5.22 (16.8)	2.77 (1.44)	1.30 (1.44)	1.41 (1.49)
Vomiting	495 (64.1%)	289 (60.0%)	36 (70.6%)	221 (59.7%)	133 (54.7%)	156 (65.3%)
Maximum vomiting episodes in 24 h median [range]	4.00 [0, 20.0]	4.00 [0, 19.0]	4.00 [0, 10.0]	4.00 [0, 19.0]	3.00 [0, 18.0]	4.00 [0, 19.0]
Duration of vomiting in days; mean [SD]	1.41 (1.42)	1.35 (1.47)	1.94 (2.05)	1.34 (1.39)	1.30 (1.44)	1.41 (1.49)
Severe dehydration (> 6% loss body weight)	128 (16.6%)	82 (17.0%)	10 (19.6%)	63 (17.0%)	50 (20.6%)	32 (13.4%)
Fever at admission	462 (59.8%)	298 (61.8%)	33 (64.7%)	223 (60.3%)	141 (58.0%)	157 (65.7%)
Vesikari score median [range]	11.0 [3.00, 20.0]	10.0 [3.00, 20.0]	12.0 [5.00, 20.0]	10.0 [3.00, 17.0]	10.0 [3.00, 17.0]	11.0 [5.00, 20.0]
Weight-for-age z score mean [SD]	− 1.72 (1.66)	− 1.69 (1.61)	− 3.03 (1.99)	− 1.52 (1.47)	− 2.03 (1.59)	− 1.33 (1.56)
Height-for-age z score mean [SD]	− 1.94 (2.45)	− 2.13 (2.52)	− 2.46 (2.50)	− 2.29 (2.51)	− 4.12 (1.50)	− 0.113 (1.55)
Mid upper arm circumference Z score mean [SD]	− 0.852 (1.28)	− 0.869 (1.35)	− 3.37 (1.53)	− 0.524 (0.879)	− 0.965 (1.13)	− 0.758 (1.56)
Rotavirus vaccine received (at least 1 dose)	658 (85.2%)	437 (90.7%)	44 (86.3%)	346 (93.5%)	233 (95.9%)	204 (85.4%)
Mothers education: primary school and below	213 (27.6%)	130 (27%)	17 (33.3%)	97 (26.2%)	80(32.9%)	50 (20.9%)
Access to improved main water source**	768 (99.5%)	478 (99.2%)	50 (98.0%)	368 (99.5%)	231 (99.1%)	237 (98.2%)

Demographic and clinical characteristics of under-five children hospitalized with diarrhea enrolled in GPDS from India for the year 2019

*Dysentery is defined as presence of blood in stools

**Improved water sources include piped water, public taps, tube wells, rainwater, covered wells, protected springs, and bore holes. Unimproved water sources were open wells, surface water, unprotected springs, bought water

### Etiology of diarrheal hospitalizations among children under five years

The five most common frequently detected pathogen in our study were adenovirus 40/41 (238/482; 49.4%), *Shigella* (117/482; 24.3%), sapovirus (103/482; 21.4%), rotavirus (81/482; 16.8%), and norovirus GII (79/482; 16.4%). Only 25 (5.2%) of the 482 children tested by qPCR had dysentery. Of these, 15 (60.0%) had Shigella detected in comparison to 102 of 457 (22.3%) without dysentery. Because dysentery was relatively rare, we did not have sufficient data to evaluate an association between Shigella quantity and dysentery.

**Fig. 1:**
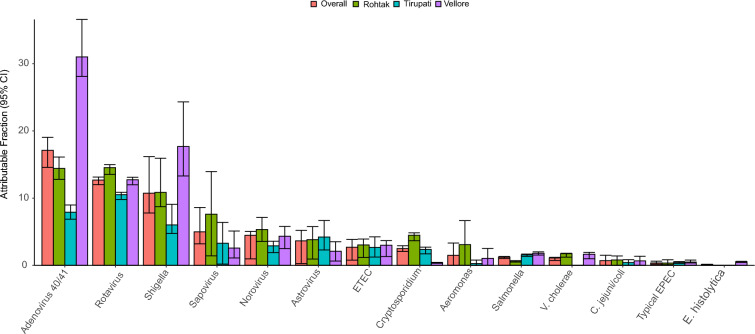
Pathogen-specific attributable fractions of under-five children hospitalized with diarrhea in India (both overall and by sentinel sites). Error bars show 95% *CI. ETEC*  enterotoxigenic *E coli.* *EPEC*  enteropathogenic *E coli*

Overall, adenovirus 40/41 was the leading cause of diarrhea (Attributable fraction [AF] 16.9%; 95% CI 14.1, 19.2) followed by rotavirus (AF 12.6%; 95% CI 11.8, 13.1), *Shigella* (AF 10.9%; 95% CI 8.4, 16.4), sapovirus (AF 4.7%; 95% CI 0.1, 9.2), norovirus (AF 4.1%; 95% CI 1.6, 5.9) and astrovirus (AF 3.4%; 95% CI 0.2, 5.5). There was some variability in the top diarrheal pathogens across the three sentinel sites, as adenovirus 40/41 was the leading pathogen in Rohtak (AF 14.7%; 95% CI 12.2, 16.9), and Vellore (AF 30.8%; 95% CI 25.9, 35.1), while rotavirus was the top pathogen in Tirupati (AF 10.4%; 95% CI 9.7, 10.9). *Shigella* was the second common pathogen in Vellore (AF 17.7%; 95% CI 13.4, 24.6), and the third commonest pathogen in Rohtak (AF 10.6%; 95% CI 7.9, 16.1), and Tirupati (AF 5.8%; 95% CI 4.2, 9.4). The site-specific and overall pathogen-attributable fractions are shown in Fig. 1.

**Fig. 2:**
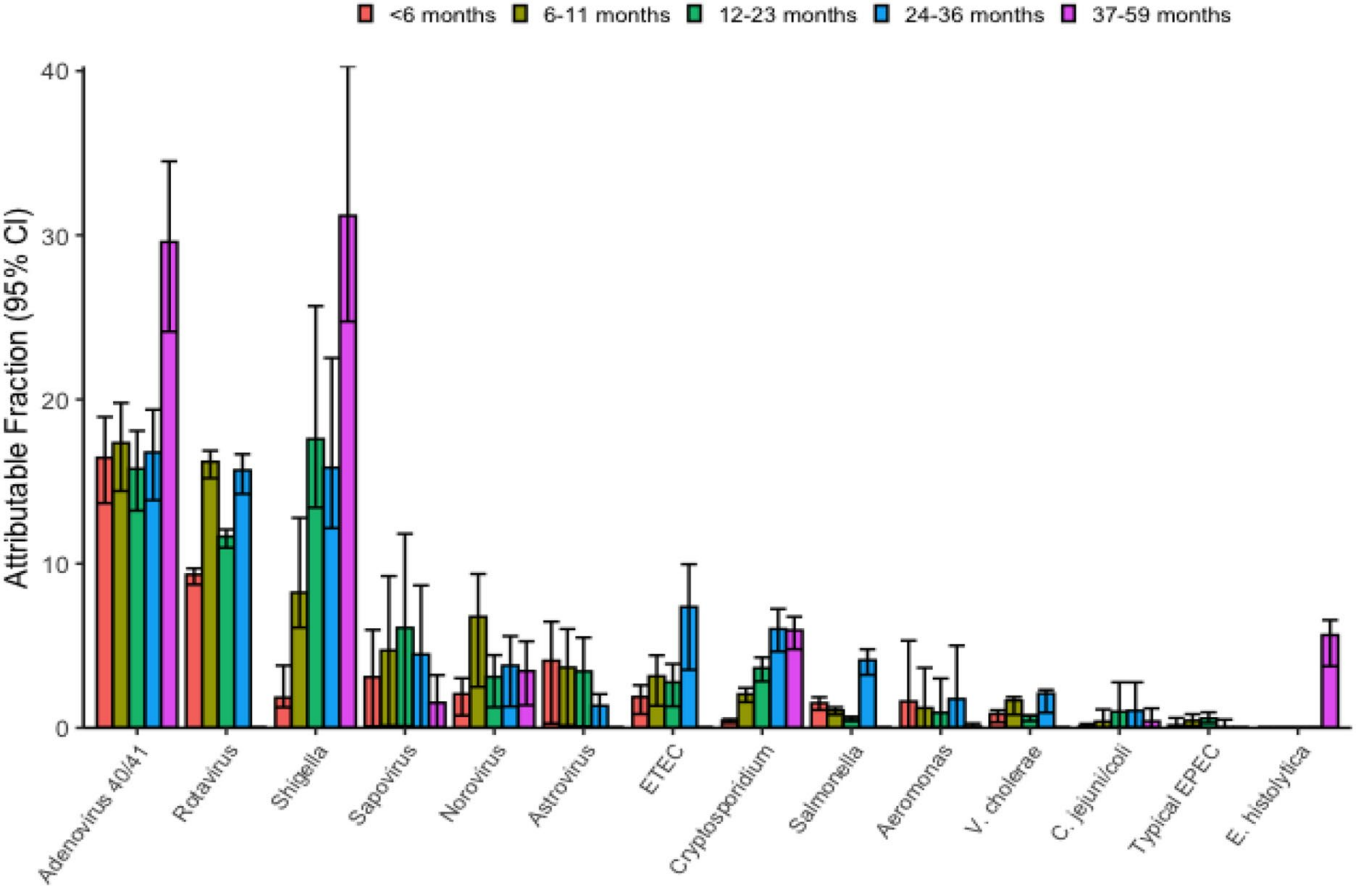
Pathogen specific attributable fractions of diarrheal hospitalizations by age in under-five children in India for the year 2019. Error bars show 95% *CI. ETEC* enterotoxigenic *E coli.* *EPEC *enteropathogenic *E coli*

Among infants, adenovirus 40/41 was the leading pathogen (AF of 16.4% in < 6 months and 17.3% in 6–11 months). Rotavirus was the second most common pathogen in infants (9.3% in children < 6 months and 16.2% in 6–11 months). Among children aged > 12 months, *Shigella* was the leading pathogen, with AF of 17.6% in 12–23 months, 15.8% in 24–36 months and 31.2% in 37–59 months of age. Enterotoxigenic *E.coli* and *Cryptosporidium* were the other major pathogens associated with diarrhea among children aged > 12 months. The pathogen-attributable fractions by age in under-five children are shown in Fig. 2.

### Association of malnutrition and diarrhea

Of the 482 samples tested by qPCR, 243 (50.4%) was from children with chronic malnutrition and 239 (49.6%) from children without chronic malnutrition. 51 samples (12.1%) included in testing were collected from acutely malnourished children among a total of 421 children for whom acute malnutrition status was available. Attribution of diarrhea to specific etiologies was not significantly associated with either acute or chronic malnutrition. (Table 2).

**Table 2:** Association of diarrheal etiology with acute and chronic malnutrition

	Acute malnutrition (Population AF) %	No acute malnutrition (Population AF)	Odds Ratio (95% CI)	Chronic malnutrition (Population AF) %	No chronic malnutrition (Population AF)	Odds ratio (95% CI)
Rotavirus	10.0	13.8	0.42 (0.14, 1.31)	13.2	12.4	1.14 (0.60, 2.18)
Shigella	16.9	12.6	1.29 (0.37, 4.51)	10.2	14.1	1.17 (0.48, 2.81)
Adenovirus 40/41	19.8	15.7	0.58 (0.13, 2.65)	12.6	20.3	0.59 (0.22, 1.59)
Norovirus	4.2	4.6	0.2 (0.02, 2.74)	4.1	4.5	0.96 (0.19, 4.89)
Sapovirus	5.1	5.1	0.7 (0.05, 10.81)	5.1	4.5	1.71 (0.28, 10.51)
Astrovirus	4.4	3.4	1.4 (0.14, 14.42)	3.9	3.1	1.19 (0.24, 5.90)
Cryptosporidium	1.5	3.3	0.2 (0.01, 7.21)	3.1	2.4	1.89 (0.32, 11.17)
ETEC	3.0	3.8	0.41 (0.03, 6.39)	2.9	4.1	0.39 (0.06, 2.55)
Bacterial etiology	22.1	21.5	0.66 (0.26, 1.72)	17.4	23.2	0.90 (0.48, 1.69)
Viral etiology	43.4	42.6	0.48 (0.21, 1.11)	38.7	44.9	0.98 (0.60, 1.62)
Protozoal etiology	1.6	3.5	0.17 (0.01, 5.22)	3.2	2.8	1.49 (0.27, 8.18)
No pathogens attributed	41.6	43.9	1.88 (0.75, 4.67)	49.1	41.0	1.08 (0.63, 1.85)

*ETEC*   enterotoxigenic E coli

Odds ratios were estimated by fitting a logistic regression with an outcome of nutritional status and proportion attributed to each etiology and etiology category as predictors, adjusted for age, gender and sentinel site

Table 2: Odds ratios were estimated by fitting a logistic regression with an outcome of nutritional status and proportion attributed to each etiology and etiology category as predictors, adjusted for age, gender and sentinel site. ETEC = enterotoxigenic E coli.

Acutely malnourished children were more likely to be lethargic at the time of hospital admission (OR: 2.48; 95% CI 1.31, 4.71). They also had a higher Vesikari score in comparison to better nourished peers, which was driven by a longer duration of diarrhea, longer vomiting duration and severe dehydration in this group. However, chronic malnutrition was not associated with any of these clinical features. Table 3 summarises the association of malnutrition with severity of diarrhea.

**Table 3:** Association of malnutrition on severity of diarrhea

	Acute malnutrition	Chronic malnutrition
Vesikari score	1.1 points (95% CI 0.34, 1.85)	− 0.07 points (95% CI − 0.51, 0.37)
Diarrheal duration	1.73 days (95% CI 0.46, 2.99)	0.36 days (95% CI -0.3, 1.03)
Maximum diarrheal episodes in 24 h	0.15 episodes (95% CI − 0.78, 1.09)	− 0.44 episodes (95% CI − 0.97, 0.09)
Maximum vomiting episodes in 24 h	0.98 episodes (95% CI − 0.04, 1.99)	− 0.1 episodes (95% CI − 0.68, 0.48)
Vomiting duration	0.5 days (95% CI 0.14, 0.86)	− 0.14 days(95% CI − 0.34, 0.06)
Vomiting	Odds Ratio 1.07 (95% CI 0.95, 1.19)	Odds ratio 0.98 (95% CI 0.92, 1.05)
Fever	Odds Ratio 1.03 (95% CI 0.91, 1.16)	Odds ratio 1.02 (95% CI 0.95, 1.1)
Severe dehydration	Odds Ratio 2.32 (95% CI 1.19, 4.53)	Odds ratio 1.47 (95% CI 0.98, 2.21)
Lethargic at admission	Odds Ratio 2.48 (95% CI 1.31, 4.71)	Odds ratio 1.5 (95% CI 1, 2.25)

Nutritional status was the predictor (with the absence of the nutritional state used as the reference group), with odds ratios estimated for dichotomous characteristics and coefficients estimated for continuous characteristics

Table 3: Nutritional status was the predictor (with absence of the nutritional state used as the reference group), with odds ratios estimated for dichotomous characteristics and coefficients estimated for continuous characteristics.

## Discussion

We examined the pathogenic profile of diarrhea among under-five children hospitalized with diarrhea, three years after the nationwide rotavirus vaccine introduction in India. A high diarrheal burden was attributed to viral agents in our population, especially in children aged < 2 years, in contrast to a few Indian studies reporting bacteria as the major contributor [13–15]. In previous studies, the most prevalent bacteria varied across studies, which is likely to be due to the different diagnostic tests used, many focusing only on a single pathogen or group of pathogens. Our study used a highly sensitive molecular method to detect a broad array of enteropathogens and hence provides a more complete picture of childhood diarrheal etiology in our settings.

When assessed by age strata and study site, the pathogens with the highest attributable fractions (AFs) were in descending order, adenovirus 40/41, rotavirus, *Shigella* spp., sapovirus, norovirus and astrovirus. Similar topmost pathogens were observed in previous multicentric studies conducted across Asia and Africa [16, 17], and in global-level results from the GPDS network in 2017–2018 [11], though with notable regional variability in rank order. The AF of rotavirus was 30.7% in Indian children in the Global Enterics Multicenter Study (GEMS) conducted prior to rotavirus vaccination and 12.7% in our study (post-rotavirus vaccine era), demonstrating the impact of rotavirus vaccines. In spite of this reduction in the burden, rotavirus still remains the second most common cause of childhood diarrhea in this cohort. Rotavirus vaccines are known to underperform in low-middle-income countries (LMICs) which explains this continued high prevalence [18]. Studies are currently underway investigating methods to improve the efficacy of the available oral rotavirus vaccines, including the introduction of birth doses or booster doses, and the development of parenteral vaccines, which may further decrease the rotavirus burden.

Many Western countries have reported the emergence of caliciviruses (norovirus and sapovirus) after the scale-up of rotavirus vaccines [8, 19]. However, in our study adenovirus 40/41 was the leading cause of diarrhea, as reported from other LMIC countries [10, 20]. A 2016 study from Northeast India also reported adenovirus as the major contributor to childhood diarrheal illness [21]. As with rotavirus, improved hygiene and sanitation alone may not suffice to combat adenovirus diarrhea [22]. Our data corroborate the current evidence favoring the development of vaccines for adenovirus 40/41 in order to alleviate the impact of pediatric diarrheal disease in LMICs though challenges related to the underperformance of oral vaccines are anticipated in these settings [23, 24].

Globally *Shigella* is the leading cause of diarrheal deaths after rotavirus [25]. In our study, *Shigella* was the most common bacteria detected accounting for nearly 47% of diarrhea in children above 2 years. Shigella infections not only pose immediate health risks, but also can have long-term effects, such as malnutrition and negative effects on physical and cognitive development [26, 27]. For these reasons, burden of diarrhea is underestimated when only incidence and mortality is accounted for and the diarrheal associated Disability-Adjusted Life Years (DALYs) increased by about 40% when long term sequelae were also taken into consideration [28]. The longitudinal analysis of the MAL-ED cohort revealed that *Shigella* had a substantial negative association with linear growth [29]. In light of the high prevalence, potential long-term impact on linear growth, and the threat of antimicrobial resistance, *Shigella* vaccines are being developed [30]. Our findings showing a significant *Shigella* burden support current efforts to develop and evaluate *Shigella* vaccines. The availability of new vaccine platforms could potentially catalyze the development of combination vaccines against the top enteropathogens. However, we need to fill the knowledge gaps in local burden data, circulating genotypes, infection, and re-infection patterns with improved molecular diarrheal surveillance to inform policymakers for decision-making around potential new enteric vaccines. Continued efforts to improve sanitation and hygiene are the key to fighting diarrheal diseases until we have improved cost-effective vaccines suiting our needs.

Childhood malnutrition is a public health concern in India. Acutely malnourished children had severe diarrhea in comparison to their well-nourished peers in our study. They had a prolonged duration of diarrhea and vomiting with severe dehydration at the time of admission. Low body weight regardless of age is reported as a risk factor for severe dehydrating diarrhea in previous studies [31–33]. The severity can be also due to (1) specific diarrheal pathogens, (2) altered gut structure, function and immune response causing prolonged recovery, and (3) social factors leading to the delayed seeking of health care [34, 35]. We did not identify any specific enteric pathogens associated with diarrhea among acutely malnourished children. A prospective study from Kenya also failed to identify specific pathogens among wasted children [31]. In other studies, pathogens like *Shigella*, campylobacter, diarrheagenic *E.coli,* and *Cryptosporidium*, have been associated with diarrhea in acutely malnourished children [36–39]. However, these studies did not examine the temporal association of the enteropathogens with malnutrition and hence it is unclear whether was a causal association or prolonged pathogen shedding, which is common among malnourished children [40, 41]. Several studies indicate a rise in morbidity and mortality associated with diarrhea in malnourished children, irrespective of the specific causative pathogen [31, 42]. This underscores the importance of prioritizing interventions to enhance the nutritional status of children, aiming to reduce both morbidity and mortality related to diarrheal illnesses.

In our study, many cases of diarrhea in acutely malnourished children did not have any identified etiology. Diarrhea not associated with a pathogen may be related to the altered gut structure, and function leading to increased intestinal permeability in acutely malnourished children [4] or might be due to a pathogen not included in our assay, despite the TaqMan Array Cards (TAC) being designed to identify a broad range of common pathogens. Another explanation could be that these children have diarrhea at low levels of pathogen replication and hence the pathogen quantity in stools could have been below the limits of detection. Since wasting is a key predictor of diarrheal mortality, functioning both as a risk factor and a consequence of diarrhea, continuous efforts focused on improving children’s nutritional status are crucial to bring down diarrhea associated mortality. Our study did not find any association between clinical severity or presence of any specific pathogen with chronic malnutrition, as has been previously reported from other African and Asian countries [31, 33, 39].

Our study had other limitations. As India is large and diverse, results from three sentinel sites may not be generalizable to the whole country. The reliability of mid-upper arm circumference (MUAC) measurements obtained during the acute diarrheal episode is another limitation. Though more resilient to dehydration than weight, MUAC can still decrease with severe dehydration. The small sample size could be a limiting factor to derive statistically significant pathogen-specific associations with malnutrition, particularly for acute malnutrition. There is a potential for underestimating bacterial pathogens in our study due to the likelihood that many children may have received antibiotics during the episode of diarrhea. Nonetheless, we consider this impact to be minimal, given our exclusion of children referred from other hospitals and our consistent policy of collecting stool samples before initiating antibiotic treatment.

## Conclusion

The burden of diarrhea still remains high in India, especially among children less than 2 years. Acutely malnourished children are more likely to develop severe diarrhea which appears to be not driven by any specific pathogen. The rotavirus burden is substantial in spite of nationwide vaccination which emphasizes the need to focus on strategies to improve the performance of rotavirus vaccines. Continued diarrheal surveillance is essential to generate data on burden and epidemiology of enteropathogens which will inform policymakers on prioritizing interventions.

## Methods

### Data and sample collection

Hospital-based diarrheal surveillance was conducted across 3 sites in India [Christian Medical College -Vellore, Pandit Bhagwat Dayal Sharma Post Graduate Institute.

of Medical Sciences, Rohtak and Sri Venkateswara Medical College, Tirupati] from January 2019—December 2019 as a part of Global Paediatric Diarrhea Surveillance (GPDS) coordinated by the World Health Organisation. All children under five years of age who were hospitalized with diarrhea were enrolled after consent from the parent/caregiver. Diarrhea was defined as three or more loose stools in a 24 h time period. Children referred from another hospital and those who developed diarrhea during hospitalization were excluded. Detailed clinical, sociodemographic and anthropometric data were collected at enrolment. The diarrheal severity was calculated using Vesikari clinical severity scoring system [43]. The stool sample was collected within 48 h of admission from all enrolled cases and was stored at -80˚C until testing.

### Sample selection for molecular testing

A subset of stool samples collected from the enrolled children was tested for enteropathogens by quantitative PCR using custom designed TaqMan Array cards. 300 samples were tested initially as a part of GPDS, where 100 samples were randomly selected from each of the 3 sentinel sites. As surplus TAC cards were available, an additional 182 samples were selected randomly such that the number of children with and without malnutrition was almost equal for the final analysis. We used stunting as the main marker of malnutrition as weight is inaccurate with dehydration. Stunting (chronic malnutrition) was defined as HAZ score < − 2 which includes both moderate stunting (− 3 < HAZ < − 2) and severe stunting (HAZ < − 3). Wasting (acute malnutrition) was defined as mid-upper-arm-circumference (MUAC) Z score of < − 2. As the mid-upper arm circumference was not available, acute malnutrition status was not defined for most of the children younger than 6 months.

### Laboratory testing

Samples were tested using custom-designed TaqMan Array Cards (Thermo Fisher, Waltham, Massachusetts, USA) as previously described [11, 44]. All the samples were tested at Christian Medical College, Vellore. Briefly, the total nucleic acid was extracted with the Qiagen QIAamp Fast DNA Stool Mini kit (Qiagen, Hilden, Germany) using a modified protocol involving bead beating. All samples were spiked with Phocine herpes virus and MS2 phage to be used as external controls for DNA and RNA, respectively, and an extraction blank was included in each extraction batch to monitor for contamination. Detections with a quantification cycle (Cq) value less than 35 were considered positive. Negative results were valid only when the corresponding external control was positive, while positive results were valid only when the corresponding extraction blank was negative for the target.

### Statistical analysis

We first calculated population attributable fractions for all children, by site, and by age. To ensure the samples tested were representative of all enrolled cases, we applied inverse probability of selection weights by fitting a logistic regression model with selection of qPCR testing as the outcome and age, seasonality of enteropathogens, site of sample collection and interaction between these factors as the covariates. Weights were then calculated as the inverse of the model-predicted probability. We used the data from Global Enterics Multicenter Study (GEMS), a case–control study that was conducted in 7 Asian and African countries, to attribute diarrhea etiology to specific enteropathogens based on the pathogen quantity detected by qPCR [16]. Using the conditional logistic regression model for GEMS, a model was fit for each pathogen to describe the association between pathogen quantity and diarrhea, with a random slope for the site to allow for variation in the strength of association between pathogen quantity and diarrhea. Pathogen quantity was defined as the log10 increase in pathogen quantity above the analytical cut-off based on the C_q_, namely $35- \frac{{\text{Cq}}}{{\text{log}}2(10)}$. A weighted population AF for each pathogen was then calculated for any stratum of *j* cases as ${\text{AF}}=1-\frac{ \sum_{1}^{{\text{j}}}\frac{{{\text{wt}}}_{i}}{{{\text{OR}}}_{i}}}{ \sum_{1}^{{\text{j}}}{{\text{wt}}}_{i}}$, where wti is the episode-specific inverse probability weight, and OR_i_ is the episode-specific and quantity-specific OR derived from the regression model [45]. The uncertainty from the AF estimates was propagated by stimulating 1000 new OR estimates from a normal distribution with the mean derived from the model co-efficient and variance–covariance from the covariate matrix.

To assess whether attribution of diarrhea to specific pathogens and pathogen types was associated with nutritional status, we fit two logistic regression models for the outcome of acute malnutrition and two additional models for the outcome of chronic malnutrition. The predictors were either the amount of attribution to individual pathogens or to pathogen categories (bacteria, viruses, protozoa, and the absence of an etiology defined as the proportion of each episode that was not attributed to a pathogen), and all models were adjusted for age, gender, and sentinel site. Next, to assess whether the phenotype of diarrhea differed by nutritional status, we fit logistic (for dichotomous outcomes) and linear regression (for continuous outcomes) models for each specific clinical characteristic, with nutritional status as a predictor and adjusted for age, gender, and sentinel site.

## Data Availability

The de-identified data will be made available from the corresponding author on reasonable request.

## Abbreviations

UIP: Universal Immunization Program
MUAC: Mid-upper-arm-circumference
GEMS: Global enterics multicenter study
MAL-ED: Malnutrition and enterics study
qPCR: Quantitative polymerase chain reaction
AF: Attributable fraction
GPDS: Global paediatric diarrhea surveillance
TAC: TaqMan array cards
